# Assessment of Cellular Apoptosis Induced by Hydroxychalcones in MCC13 Merkel Cells

**DOI:** 10.3390/ijms27114897

**Published:** 2026-05-28

**Authors:** Marcelina Chmiel, Aleksandra Włoch, Natalia Potocka-Wojtowicz, Monika Stompor-Gorący

**Affiliations:** 1 Department of Organic Chemistry, Faculty of Medicine, University of Rzeszów, 35-310 Rzeszów, Poland; 2 Department of Physics and Biophysics, Wrocław University of Environmental and Life Sciences, 50-375 Wrocław, Poland; aleksandra.wloch@upwr.edu.pl; 3 Department of General Genetics, Faculty of Medicine, Collegium Medicum, University of Rzeszów, 35-310 Rzeszów, Poland; npotocka@ur.edu.pl; 4 Department of Pathophysiology, Faculty of Medicine, University of Rzeszów, Rejtana, 35-310 Rzeszów, Poland

**Keywords:** hydroxychalcones, annexin V, mitochondrial membrane potential, oxidative stress, Merkel cell carcinoma

## Abstract

Chalcones, as flavonoid precursors, are known to have biological importance and vast pharmacological effects. These bioactive molecules have been used in traditional medicine for many years for treatment of various diseases, particularly because of their antitumor activity. The aim of this study was to assess antiproliferative activity of selected hydroxychalcones against six cancer cell lines, derived from various human organs and with varying degrees of aggressiveness and resistance to cytostatics (5637, A-431, UM-SCC-17A, SK-MEL-3, MCC13, A172) in comparison to two noncancerous cell lines (MCF-10A and BALB/3T3). The specific goal of the present study was to assess the influence of 2′-hydroxychalcone, 4-hydroxychalcone and 4′-hydroxychalcone, the compounds with the same molar mass but with different positions of the hydroxyl group, on different stages of apoptosis. Additionally, we aimed to determine the involvement of mitochondria in the initiation of the cell death process. The principal cell line used for validation was Merkel cell carcinoma, a rare type of human skin cancer. The aforementioned compounds were supplemented for 24 and 48 h at several concentrations. In this paper we demonstrate the ability of hydroxychalcones to activate an early stage of apoptosis by exposure of phosphatidylserine on the cell surface with simultaneous changes in mitochondrial membrane. Our results acknowledge and strengthen the prospect of using chalcones for development of new therapeutic strategies in various oncological disease models.

## 1. Introduction

Chalcones (1,3-diaryl-2-propen-1-ones) are a group of organic compounds in which two aromatic rings, A and B, are linked to each other by the carbonyl group conjugated with an α,β-unsaturated C=C double bond. These acetophenone derivatives are intermediate products in biosynthesis of natural flavonoids. Their characteristic feature is an open heterocyclic ring, the closure of which transforms a chalcone into a flavanone. In nature, chalcones are found either in the free form, as aglycones, or in the form of glycosides or esters. They are found in such plants as *Glycyrrhiza glabra* [1], *Humulus lupulus* [2], *Helichrysum arenarium* [3], *Salix* sp. [4] and many others. Natural chalcone precursors, dihydrochalcones, are known for their sweet taste and a range of health-promoting properties [5]. Through chemical synthesis, chalcones are obtained, among others, by Claisen–Schmidt condensation under basic conditions [6]. 2′-Hydroxychalcones can also be obtained by biotechnological methods, such as microbial biotransformations [7,8]. Moreover, chalcones often serve as substrates for synthesis of new derivatives [9], including flavanones [10], which have anti-inflammatory activity. Both natural and synthetic chalcones show a broad spectrum of pharmacological activity, such as antimicrobial activity [11,12], anticancer activity [13,14,15] (including antimetastatic activity [16]), antifungal activity [17,18], proapoptotic activity [19], neuroprotective activity [20], antiarrhythmic activity [21], alleviation of the symptoms of metabolic syndrome [22], anti-angiogenic activity [23] and antiviral activity [24,25]. Numerous chalcones bearing hydroxyl groups are continuously explored for their ability to regulate the expression of inflammatory cytokines and enzymes associated with inflammatory disorders [26,27,28,29]. For instance, 2′-hydroxychalcone inhibited the expression of ICAM-1, VCAM-1 and E-selectin in a concentration-dependent manner in primary endothelial cells with simultaneous inactivation of nuclear factor-kB (NF-kB) [30]. Moreover, 4′-hydroxychalcone demonstrated inhibitory effects on TNFα-induced NF-κB activation, primarily through proteasome inhibition [31] and its capability of blocking the nuclear translocation and DNA binding of the NF-κB subunit RelA [32]. For this reason, chalcones are of great interest as anti-inflammatory agents.

Mitochondrial dysfunction plays a critical role in the pathogenesis of numerous serious diseases, owing to its impact on cellular energy metabolism, oxidative stress, and apoptotic signaling. Therefore, identifying compounds that can effectively target mitochondrial malfunction is pivotal for development of therapeutic strategies to overcome these challenges. In a high-throughput phenotypic assay designed to evaluate effects on mitochondrial morphology and health in human neurons derived from induced pluripotent stem cells (iPSCs), treatment with 4-hydroxychalcone resulted in significantly increased mitochondrial length and decreased mitochondrial circularity compared with untreated controls [33]. In addition, this compound increased mitochondrial reactive oxygen species in a mammalian cell culture, giving rise to increased turnover of mitochondrial protein frataxin (FXN) in Friedreich’s ataxia, which can delay progression of this disease [34]. Furthermore, 4′-hydroxychalcone was found to impair respiratory control and oxidative phosphorylation, indicating a disturbance in mitochondrial energy metabolism. What is more, 4′-HC stimulated ATPase activity and induced rapid potassium release from mitochondrial vesicles, resulting in increased mitochondrial membrane permeability [35]. The results demonstrate that structurally related compounds can disrupt mitochondrial energy transfer and membrane integrity, supporting the idea that these chalcones affect fundamental mitochondrial functions.

Moving forward, cancer cells are suppressed mainly through ROS-mediated mitochondrial cell death, Akt/mTOR and FAK pathways [36], G2/M cell cycle arrest, DNA damage, apoptosis, and modulation of MAP kinase activity [37]. According to De Moura Escobar et al. [38], 4′-hydroxychalcone decreases the viability of neuroblastoma SH-SY5Y cells, promotes alteration in cell cycle progression causing cellular morphological changes, ROS increase and mitochondrial alterations. It was found that 2′-hydroxychalcone, in a dose-dependent manner, reduced the viability of lipid-loaded HepG2 cells and increased the level of LDH released from the lipid-loaded hepatocytes, suggesting the hepatotoxic potential of this compound [39]. The copper (II) complexes involving 2′-hydroxychalcone derivatives demonstrated high cytotoxicity against 10 human cancer cell lines. For some of the complexes, intercalation into DNA was observed, similar to the effect demonstrated by ethidium bromide [40]. Moreover, 2′-hydroxychalcones belong to the group of autophagy inducers in cancer cells, e.g., the A549 cell line and others [41]. However, 2-hydroxychalcone with a hydroxyl group in the aromatic ring B induces apoptosis and necrosis in a human epidermal keratinocyte (HaCat) cell line by promoting generation of reactive oxygen species (ROS). In addition, it shows anti-dermatophytic activity against *Trichophyton rubrum* and *Trichophyton mentagrophytes* [42].

The hydroxychalcone derivatives that, apart from the OH group, have other additional substituents, such as methyl, acyl, nitro or halogen atoms, are also successively investigated [43]. For instance, it was demonstrated that the chalcone derivative 3′-methyl-3-hydroxychalcone inhibited proliferation of various types of cancer cells, including HGC-27 (gastric cancer), HeLa (cervical carcinoma), PANC-1 (pancreatic cancer) and GOTO (neuroblastoma). These occur as a result of the cell cycle disruption and changes in phosphorylation of some proteins. Additionally, 3′-methyl-3-hydroxychalcone suppressed the promoting activity of 12-O-tetradecanoylphorbol-13-acetate on skin carcinogenesis in 7,12-dimethylbenz[a]anthracene-initiated mice [44]. A fluorine derivative (4-fluorochalcone) exerted a significant inhibition effect (IC_50_ 9.49 μM) on human glutathione S-transferase [45], which plays a major role in detoxification of chemotherapeutic agents. Many studies on 4′-(p-toluenesulfonylamide)-4-hydroxychalcone (TSAHC) demonstrated its antitumorigenic effect on hepatocarcinoma [46,47,48], inhibitory activity towards cytochromes [49,50], suppressive effects on rheumatoid arthritis [51] and depigmentation properties by inhibiting tyrosinase catalytic function [52]. Another synthetic chalcone derivative, 4′-acetamide-4-hydroxychalcone, reduced vascular endothelial growth factor-induced migration and invasion of glioma cells (U87-MG) both in vitro and in vivo in a xenograft model [53]. Additionally, introducing a chlorine atom at the C4 position in 2′-hydroxychalcone gave the compound that exhibited a high selectivity towards human acetylcholinesterase, the enzyme implicated in progression of Alzheimer’s disease [54]. Similar enhancements in biological activity have also been observed with methoxy substituents, suggesting that the appropriate electron-donating or halogen functionalization plays a significant role in modulating enzyme–ligand interactions [55].

In our previous study we investigated the influence of xanthohumol and its derivatives on the rare skin cancer Merkel cell carcinoma. To extend that work, herein we analyzed three hydroxychalcones bearing hydroxyl groups at different positions, corresponding to the positions of hydroxyl groups in xanthohumol, for induction of apoptosis.

## 2. Results

### 2.1. Antiproliferative Activity Effect of Hydroxychalcones on Selected Cell Lines

The chalcone derivatives containing hydroxyl groups were evaluated for their ability to inhibit cell proliferation in vitro. Table 1 lists IC_50_ values experimentally determined for 2′-HC, 4-HC, and 4′-HC chalcones in six tumor cell lines with varying degrees of aggressiveness and resistance to cytostatics (5637, A-431, UM-SCC-17A, SK-MEL-3, MCC13, A172) and two non-cancerous cell lines, human MCF-10 and murine BALB/3T3.

Blader carcinoma cells (5637) were the most sensitive to 4′-hydroxychalcone with the lowest IC_50_ value (6.7 μM), whereas for SK-MEL-3 cells (melanoma cell line) the IC_50_ value was nearly two-fold higher than for A-431, BALB/3T3, and A172 cell lines (11.1, 14.4, and 16.3 μM, respectively). For Merkel cell carcinoma MCC13 cells, two hydroxychalcones (2′-HC and 4-HC) showed similar potency in inhibiting proliferation (IC_50_ values of 25.7 and 27.0 μM respectively), whereas 4′-HC demonstrated higher activity, with the IC_50_ value of 13.9 μM. Such a dependence was observed for all the tested cell lines. Cisplatin, one of the most important and widely used chemotherapy drugs, was used as reference compound. Out of all tested cell lines only the human glioblastoma cell line (A172) showed a lower IC_50_ parameter in comparison to cisplatin. The IC_50_ values obtained in the non-cancerous control cell line (MCF-10A) were in some cases higher than those observed in tumor cell lines.

The selective activity of the tested compounds was evaluated using the selectivity index (SI), calculated by comparing their cytotoxic effects in normal cell lines with those observed in cancer cells. The SI was determined as the ratio of the IC_50_ value obtained for the normal cell lines (SI_1_—MCF-10A; SI_2_—BALB/3T3) to the IC_50_ value for the corresponding cancer cell line. SI values greater than 1.0 indicate that the compound exhibits stronger activity against tumor cells than against non-cancerous cells.

The calculated selectivity indexes for the analyzed compounds are presented in Table 2.

The selectivity index values (SI_1_ and SI_2_) for a given compound vary depending on the analyzed cancer cell line, reflecting the distinct antiproliferative responses observed in the human MCF-10A and murine BALB/3T3 normal cell lines.

Selectivity index analysis comparing MCF-10A and MCC13 cells revealed differential selectivity among the tested isomers, with SI values of 0.73 for 2′-HC, 1.6 for 4-HC, and 1.7 for 4′-HC. These results indicate that 4-HC and 4′-HC exhibited moderate selectivity toward MCC13 cells relative to non-cancerous MCF-10A cells, whereas 2′-HC demonstrated lower selectivity under the tested experimental conditions.

Among the tested cell lines, the MCC13 model, derived from Merkel cell carcinoma, was selected for further studies as a representative of a rare and clinically challenging malignancy. Although flavonoids and hydroxychalcones have demonstrated anticancer activity in various common cancer models, there is a clear lack of data regarding their effects and underlying cellular mechanisms in rare cancers, including Merkel cell carcinoma. Therefore, MCC13 was chosen as an initial proof-of-concept model to explore the potential activity of the studied compounds in this underexplored disease context. Our aim was to characterize the cellular response of the rare MCC13 cancer cells and determine whether the tested compounds are capable of inducing mitochondrial depolarization and ROS elevation in a tumor-derived model.

Based on the IC_50_ values obtained from the SRB assay, further experiments were conducted using two concentrations—above IC_50_ and beyond IC_90_ levels—to evaluate responses of apoptosis induction, mitochondrial dysfunction, and ROS generation.

### 2.2. Effect of Hydroxychalcones on Apoptosis of MCC13 Cells Evaluated by Annexin V Staining

**Figure 1:**
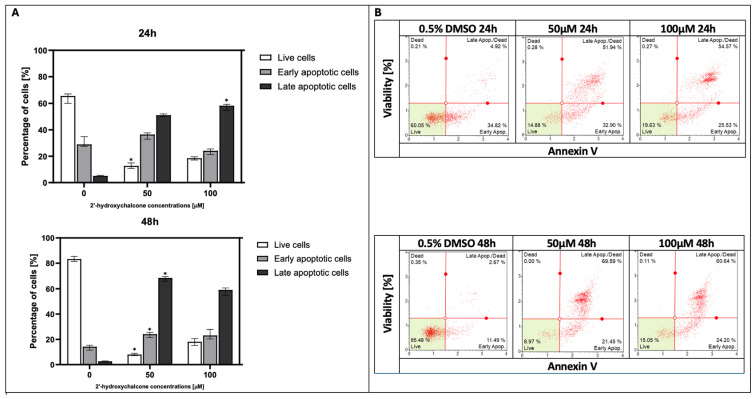
(**A**) Percentage ratio of cellular apoptosis in MCC13 cells after treatment with 2′-hydroxychalcone. Statistical significance was assessed separately for live, early-apoptotic and late-apoptotic cells using the Friedman test with post hoc test. Data are presented as medians ± SD. Significant differences between concentrations are indicated by asterisks above the corresponding bars. * *p* < 0.05 vs. control within the same cell population. (**B**) Apoptosis profile of MCC13 cells after 24 and 48 h. The plot reflects different cellular stages: live, early-apoptotic, late-apoptotic/dead cells and nuclear debris.

**Figure 2:**
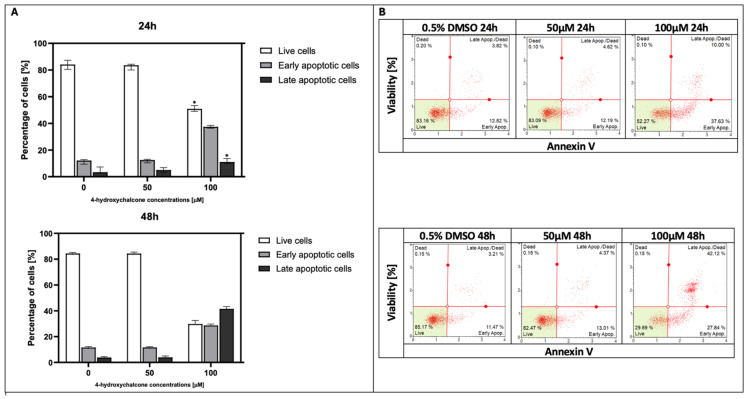
(**A**) Percentage ratio of cellular apoptosis in MCC13 cells after treatment with 4- hydroxychalcone. Statistical significance was assessed separately for live, early-apoptotic and late-apoptotic cells using the Friedman test with post hoc test. Data are presented as medians ± SD. Significant differences between concentrations are indicated by asterisks above the corresponding bars. * *p* < 0.05 vs. control within the same cell population. (**B**) Apoptosis profile of MCC13 cells after 24 and 48 h. The plot reflects different cellular stages: live, early-apoptotic, late-apoptotic/dead cells and nuclear debris.

**Figure 3:**
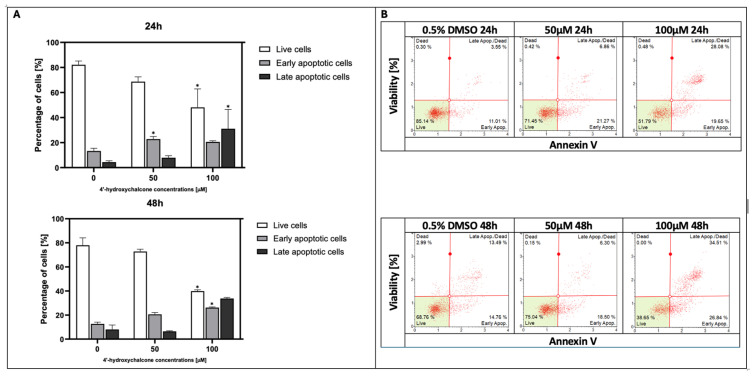
(**A**) Percentage ratio of cellular apoptosis in MCC13 cells after treatment with 4′-hydroxychalcone. Statistical significance was assessed separately for live, early-apoptotic and late-apoptotic cells using the Friedman test with post hoc test. Data are presented as medians ± SD. Significant differences between concentrations are indicated by asterisks above the corresponding bars. * *p* < 0.05 vs. control within the same cell population. (**B**) Apoptosis profile of MCC13 cells after 24 and 48 h. The plot reflects different cellular stages: live, early-apoptotic, late-apoptotic/dead cells and nuclear debris.

After 24 h of treatment with 2′-hydroxychalcone we observed a significant drop in live cell percentage, proportional to the increasing chalcone concentration. The number of cells in the early stage of apoptosis was similar at the 2′-HC concentrations of 50 and 100 μM. A significant response was noted for the cells at the late stage of apoptosis at both concentrations. Interestingly, the supplementation for 48 h did not notably change the viability of cells at any stage of apoptosis. A significant reduction in live cells was observed at the concentration of 50 μM, and likewise for the cells in the early and late stages of apoptosis, compared with the control sample (Figure 1). Similarly to 2′-hydroxychalcone, incubation with 4-hydroxychalcone for 24 h was followed by the statistically meaningful drop in the amount of non-apoptotic cells. However, the reduction in the number of viable cells was decreasing below the concentration of 100 μM, and at 50 μM the number of living cells was similar to the control. The highest concentration of the early-apoptotic cells (annexin V (+) and 7-AAD (−)) was noted for the 4-hydroxychalcone dose of 100 μM. In terms of the late stage of apoptosis, the number of cells was increased in a dose-escalating manner. Extending the incubation time to 48 h did not radically change the percentage of apoptotic cells at any stage of apoptosis. Only the cells at the last stage of apoptosis increased in number after 48 h of incubation. Similar results were observed for viable cells at the compound concentration of 50 μM and vehicle control (Figure 2). Regarding the treatment with 4′-hydroxychalcone, the percentage of viable cells did not change at the tested concentrations for 24 h. A statistically significant drop was observed only at 100 μM, yet the shrinkage was slight. The level of early-apoptotic cells was steady and identical with the late-apoptotic cells after treatment for 24 h with 50 μM dose compared to control. After 48 h of incubation, there were no significant differences between the populations compared to the previous timepoint, implicating a maintained effect of response (Figure 3). Statistical significance was noted for early-apoptotic populations between the compound concentrations of 50 and 100 μM.

### 2.3. Mitochondrial Membrane Effect of Hydroxychalcones on MCC13 Cells Assessed by Mitopotential Assay

**Figure 4:**
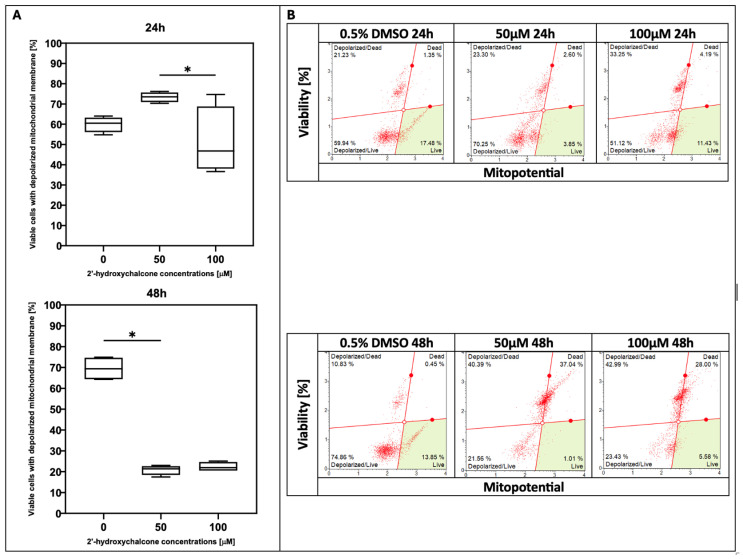
(**A**) Effect of the 2′-hydroxychalcone treatment in MCC13 cell line after 24 and 48 h. All values are presented as medians ± SD using ANOVA Friedman *p* < 0.05 and post hoc tests * *p* < 0.05. (**B**) MCC13 cells’ health profile after 24 and 48 h of treatment. Green section represents live cells, and red lines mark the area of depolarized/live cells, depolarized/dead cells and dead cells.

**Figure 5:**
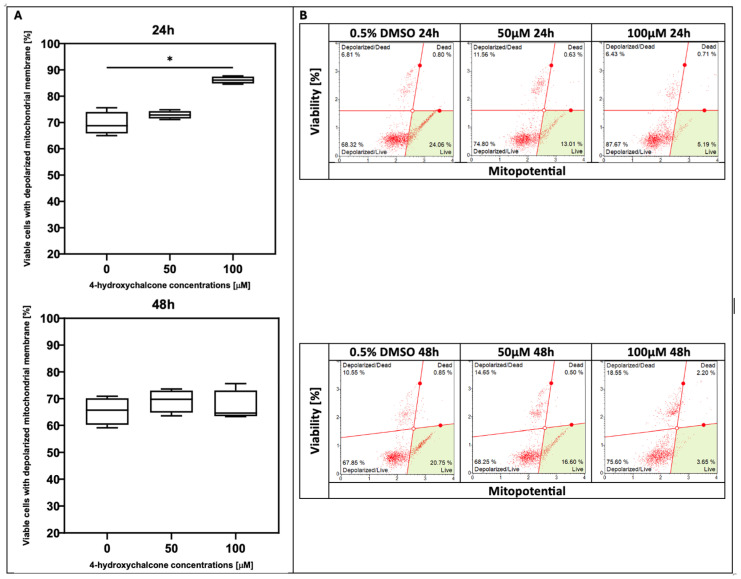
(**A**) Effect of the 4-hydroxychalcone treatment in MCC13 cell line after 24 and 48 h. All values are presented as medians ± SD using ANOVA Friedman *p* < 0.05 and post hoc tests * *p* < 0.05. (**B**) MCC13 cells’ health profile after 24 and 48 h of treatment. Green section represents live cells, and red lines mark the area of depolarized/live cells, depolarized/dead cells and dead cells.

**Figure 6:**
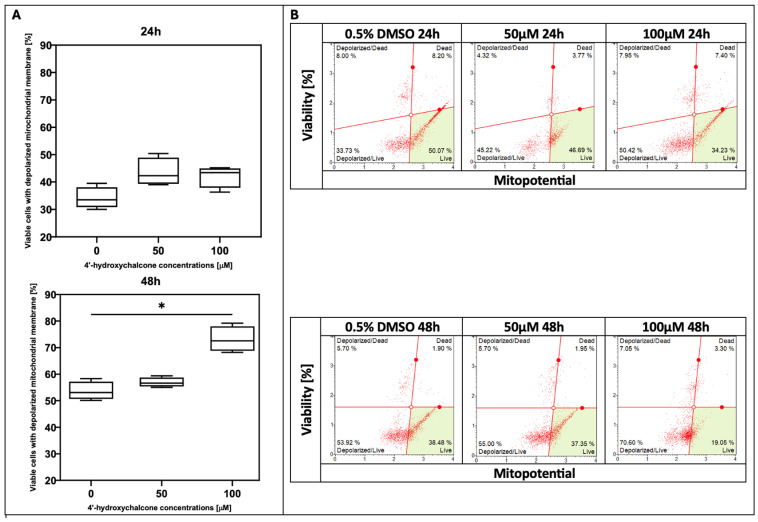
(**A**) Effect of the 4′-hydroxychalcone treatment in MCC13 cell line after 24 and 48 h. All values are presented as medians ± SD using ANOVA Friedman *p* < 0.05 and post hoc tests * *p* < 0.05. (**B**) MCC13 cells’ health profile after 24 and 48 h of treatment. Green section represents live cells, and red lines mark the area of depolarized/live cells, depolarized/dead cells and dead cells.

Supplementation with 2′-hydroxychalcone for 24 h affected the percentage of viable cells with depolarized mitochondrial membrane potential. The highest number of live cells was observed at the lowest dose (50 μM) and it was gradually dropping with increasing the dose. The effect on mitochondrial membrane was enhanced after 48 h of incubation. The percentage of live cells diminished from 70 to 20% at the dose of 50 μM, and a similar trend was observed at dose of 100 μM. Based on available cell health profiles, the percentage of dead cells (positive for 7-AAD) rises with increasing the concentration of 2′-hydroxychalcone for each timepoint (Figure 4). Regarding 4-hydroxychalcone, a significant rise in viable cells was noted at the concentration of 100 μM, although the fraction of depolarized/live cells was comparatively similar in the whole range of concentrations after 24 h. After 48 h of incubation the viability of cells with depolarized membrane potential did not change compared to the control sample; however, the percentage of dead cells with depolarized membranes was rising (Figure 5). In the case of 24 h treatment with 4′-hydroxychalcone, no significance was observed at 50 and 100 μM; however, the percentage of viable cells moderately increased compared to the control group. Extending the time of 4′-hydroxychalcone supplementation to 48 h improved cell viability and increased mitochondrial membrane potential up to 2-fold with statistical significance achievement (Figure 6).

### 2.4. Oxidative Stress Effect of Hydroxychalcones on MCC13 Cells Assessed by the Oxidative Stress Kit

**Figure 7:**
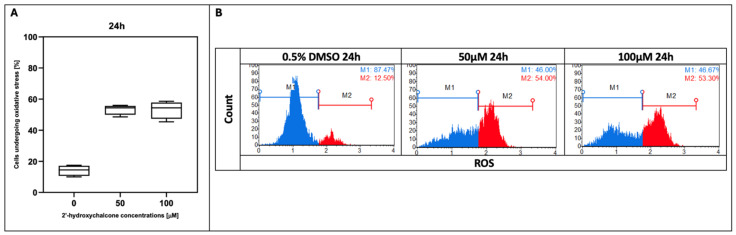
(**A**) Percentage of ROS-positive MCC13 cells after the treatment with 2′-hydroxychalcone. Data are presented as medians ± SD. No statistically significant differences were observed. (**B**) ROS profiles of MCC13 cell line after 24 h of treatment. The histogram groups two cell populations: ROS (−) (blue area) and ROS (+) (red area). Cells were analyzed by flow cytometry.

**Figure 8:**
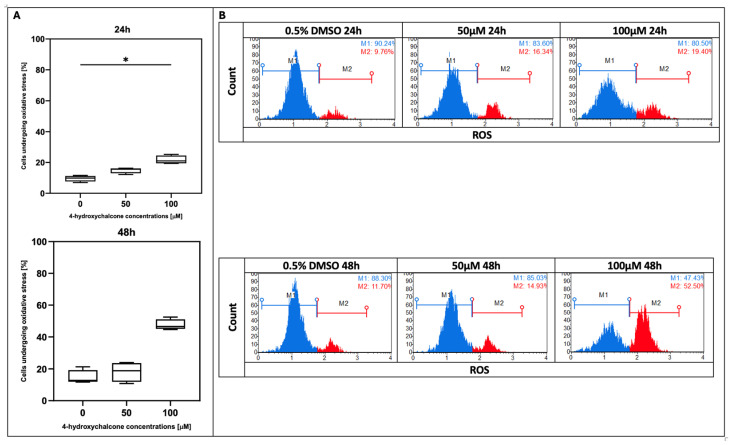
(**A**) Percentage of ROS-positive MCC13 cells after the treatment with 4-hydroxychalcone. Data are presented as medians ± SD. Statistical significance is indicated using ANOVA Friedman *p* < 0.05 and post hoc tests * *p* < 0.05. (**B**) ROS profiles of MCC13 cell line after 24 and 48 h of treatment. The histogram groups two cell populations: ROS (-) (blue area) and ROS (+) (red area). Cells were analyzed by flow cytometry.

**Figure 9:**
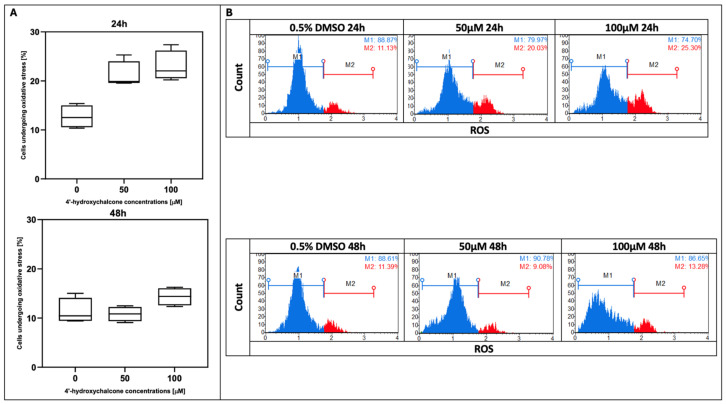
(**A**) Percentage of ROS-positive MCC13 cells after the treatment with 4′-hydroxychalcone. Data are presented as medians ± SD. No statistically significant differences were observed. (**B**) ROS profiles of MCC13 cell line after 24 and 48 h of treatment. The histogram groups two cell populations: ROS (−) (blue area) and ROS (+) (red area). Cells were analyzed by flow cytometry.

In the course of a 24 h incubation, 2′-hydroxychalcone induced generation of ROS (reactive oxygen species) in MCC13 cells in both supplemented doses; however, statistical significance was not achieved (Figure 7). This compound was not evaluated at later timepoints. Similarly to 2′-hydroxychalcone, its structural analog 4-hydroxychalcone started to trigger the production of ROS after 24 h of incubation. A concentration dependency was observed also for this compound, and a statistically detectable effect was achieved for 100 μM in comparison with DMSO control. After 48 h of the treatment only the highest tested concentration expanded the percentage of cells undergoing oxidative stress up to 50% (Figure 8). Under the treatment with 4′-hydroxychalcone no significant effect was observed for the first 24 h. The cells treated with 50 and 100 μM of 4′-hydroxychalcone had a stable level of ROS compared to the control sample. Longer incubation time (up to 48 h) appeared to have a positive effect on the cells. Reduced generation of ROS was observed at each concentration point (Figure 9).

## 3. Discussion

Available research indicates that chalcones and hydroxychalcone derivatives exhibit significant anticancer activity across multiple cancer cell lines, including A-431 and glioblastoma models, with IC_50_ values typically in the low micromolar range [56,57]. Flavonoids such as xanthohumol have been evaluated in a broader panel of cell lines, including MCC13, demonstrating moderate cytotoxicity (IC_50_ 12–32 µM), which further supports the potential of this class of compounds in rare cancer models [58]. Other natural compounds like flavokawain A [59] or isoliquiritigenin [60] have been shown to induce cell cycle arrest in high-grade bladder cancer cell lines including 5637. Although compounds that meet the international criterion of antiproliferative activity (4 μg/mL) for synthetic agents against human cancer cell lines are considered promising, the available data remain limited, particularly in studies involving rare cancer cell lines such as Merkel cell carcinoma models, for instance MCC13.

Type 1 programmed cell death, called apoptosis, is a well-known phenomenon occurring in cell membranes. Intracellular changes are related to intrinsic and extrinsic signaling pathways leading to cell death. The majority of neutral phospholipids are found on the surface of healthy cells, whereas the inner layer of the cell membrane is abundant in phosphatidylserine. Yet in the early stage of apoptosis, phosphatidylserine is translocated to the outer layer of the cell membrane, where it can be easily seized by the annexin V protein. Progressive stages of apoptosis can be detected through dual staining with 7-AAD, a fluorescent DNA intercalator that distinguishes late-apoptotic and dead cells [61,62]. Another meaningful feature that can be used to identify apoptotic cell death is dissipation of mitochondrial membrane potential. Intrinsic apoptotic signaling is associated with the loss of mitochondrial integrity, leading to the release of pro-apoptotic factors from mitochondrial membrane to the cytosol. Changes in electric charge distribution within the cell membrane interfere with electron transportation, which is followed by generation of reactive oxygen species, triggering membrane permeability transition and progression of irreversible apoptosis [63,64].

This study aimed to assess the inhibitory effect induced by three hydroxychalcones, with the same molar mass but different arrangement of hydroxyl groups (2′-HC, 4-HC, and 4′-HC), on the cell viability and stimulatory effect of these compounds on apoptosis of MCC13 cells. We showed that 2′-hydroxychalcone induced apoptosis in MCC13 cells after the treatment for 24 and 48 h. Application of 50 μM of 2′-HC leads to transition of healthy cells to late-stage apoptotic ones, compared with the vehicle control. Using a higher dose of 2′-HC intensifies this effect. To further investigate the involvement of 2′-HC in the apoptotic pathway, its effect on mitochondrial membrane potential was assessed. The results demonstrated that 2′-hydroxychalcone induced significant depolarization of the membrane after 24 and 48 h of the treatment. A substantial loss of membrane potential was observed at both tested concentrations, supporting the hypothesis that induction of apoptosis proceeds via the mitochondria-dependent mechanism. The cells treated with increasing concentrations of 2′-hydroxychalcone showed overproduction of ROS after just 24 h. In Wang’s study [65] the researchers confirmed the pro-apoptotic activity of 2′-hydroxychalcone, which induced autophagy-dependent apoptosis in the MCF-7 cell line. The Western blot analysis showed that 2′-HC triggered the cleavage of caspase-9, caspase-3, and PARP proteins and augmented the expression of pro-apoptotic protein Bax, while suppressing the expression of the anti-apoptotic protein Bcl-2. Similarly, Pawlak’s team [66] supported this characteristic by demonstrating that low doses (10 μM) of 2′-hydroxychalcone induced apoptosis and increased content of caspases in canine lymphoma and leukemia cells after 48 h of incubation. Another research group [39] confirmed that 2′-HC caused alterations in mitochondrial membrane potential in treated hepatocytes. In a lipid-loaded model this compound modified respiratory function and mitochondrial activity, reflected by changes in the membrane potential. Additionally, they evaluated the level of intracellular ROS, along with decreased antioxidant defense against oxidative stress. Research conducted by Wang et al. [65] showed that intracellular ROS in MCF-7 cells increased dramatically in dose- and time-dependent manners after the exposure to 2′-HC. Nevertheless, formation of ROS varies across tested models. In breast cancer cells (MDA-MB-231) within the concentration range of 10–40 µM, 2′-hydroxychalcone did not induce significant alterations in ROS level, compared with control cells [67]. What is more, Shen et al. [68] reported its anti-oxidative activity, as it ameliorated oxidative stress in zebrafish. The discrepancies between the results from in vitro cellular models and in vivo systems suggest that the redox-modulating effects of 2′-hydroxychalcone are selective and depend on biological complexity and experimental context.

In contrast to 2′-hydroxychalcone, its regioisomer 4-hydroxychalcone exhibited apoptosis induction at 100 µM, despite stable mitochondrial membrane potential at 24 h, with significant depolarization observed only after prolonged incubation (48 h) and higher concentrations. ROS generation was also delayed and less consistent, appearing at 100 µM after 48 h. This temporal discrepancy indicates that apoptosis may occur independently of early mitochondrial dysfunction and is not directly correlated with ROS generation at initial time points, suggesting a distinct or delayed mechanism of action. Similar conclusions were drawn by Alshangiti et al. [69], who reported that 4-hydroxychalcone significantly increased the level of reactive oxygen species (ROS) in MYCN-amplified human neuroblastoma cells after the treatment with 25–100 µM of the compound, along with decreasing cellular levels of the antioxidant glutathione. The elevated ROS level was associated with mitochondrial dysfunction and cell death, and cytotoxicity could be partially prevented by antioxidants or mitochondrial ROS scavengers, indicating that oxidative stress plays a functional role in the 4-HC-induced cytotoxicity. Of particular interest is the dual activity of 4-HC in an in vivo model. According to the study of Abdelmawgood’s group [70] on an ovalbumin-induced mouse model of allergic asthma, the treatment with 4-hydroxychalcone significantly reduced lung oxidative stress, as evidenced by decreased markers of oxidative damage. This antioxidant effect was associated with activation of the Nrf2/GPx4 signaling pathway, suggesting that 4-HC enhances endogenous antioxidant defenses in vivo. Similar conclusions were drawn by Nazar et al. [71], who observed that 4-HC attenuated the cisplatin-induced ROS production in the in vivo mice models.

It is worthwhile to mention that there is another interesting hydroxychalcone, namely 4′-hydroxychalcone. Our results give evidence that a moderate but coordinated increase in ROS levels, mitochondrial depolarization, and apoptosis was observed at 100 µM, suggesting a weaker involvement of mitochondrial pathways. These findings are supported by the correlation between oxidative stress and mitochondrial impairment. Disfunction of respiratory chain complex leads to membrane depolarization and electron leakage, causing elevated ROS levels [72]. Our results are partially consistent with those observed by De Moura Escobar [38]. Exposure of phosphatidylserine on the cell surface (a hallmark of apoptosis) was pronounced after administration of 40 μM of 4′-HC in SH-SY5Y cells. Concomitantly, they observed a meaningful increase in the percentage of cells undergoing oxidative stress (up to 70%), along with the loss of MMP (mitochondrial membrane potential) by 50%. Taking these data together, an increase in mitochondrial membrane potential suggests mitochondrial hyperpolarization, which has been reported as an early or transient event preceding apoptosis, rather than a hallmark of classical mitochondrial dysfunction.

The differences in the chemical behavior of 2′-hydroxychalcone, 4-hydroxychalcone, and 4′-hydroxychalcone result directly from the position of the hydroxyl group in relation to the α,β-unsaturated carbonyl system, which influences the molecular conformation, electron density distribution, and chemical reactivity [73]. In 2′-hydroxychalcone, the hydroxyl group is in the ortho position relative to the carbonyl bond, allowing the formation of a stable intramolecular hydrogen bond with the carbonyl oxygen. This interaction limits the conformational freedom of the molecule, increases the planar structure of the system, and modifies the polarity of the carbonyl group and the C=C bond [74,75]. As a result, 2′-hydroxychalcone exhibits increased reactivity as a Michael acceptor, which favors chemical reactivity towards nucleophiles. In terms of chalcones with a para-hydroxyl group (4-hydroxychalcone and 4′-hydroxychalcone), they differ in the aromatic ring in which the –OH group is located. In 4-hydroxychalcone, the hydroxyl group is attached directly to the ring connected to the carbonyl group, while in 4′-hydroxychalcone, the ring is separated from the carbonyl group by the C=C bond. These differences lead to subtle but significant differences in the charge distribution in the molecules, the stability of resonance forms, and the chemical reactivity of the carbonyl system. In 4-hydroxychalcone, the hydroxyl group is conjugated with the carbonyl and the C=C bond, and therefore reacts more readily with nucleophiles. However, in 4′-hydroxychalcone the –OH group is located in the ring separated from the carbonyl by the C=C bond, which provides greater stabilization of the molecule, but at the expense of reactivity [76,77,78].

According to the antiproliferative results, 4′-HC was identified as the most active compound among those tested on the cancer cell lines. However, in the subsequent analyses 2′-hydroxychalcone was revealed to have the highest activity on the Merkel cell line. Although all three compounds share the same molar mass, they differ in the position of the hydroxyl group. This seemingly minor structural variation has a pronounced impact on their biological activity, as evidenced by the effects on apoptosis induction, mitochondrial membrane potential, and ROS generation. Such variability highlights their dual and cell line-dependent behavior, underscoring their potential as valuable chemical scaffolds for studying cellular processes. Moreover, this diversity may be advantageous for the development of targeted therapeutic strategies, provided that their structure–activity relationships are carefully optimized.

## 4. Materials and Methods

### 4.1. Cell Culture and Chemicals

MCC13 cells were cultured according to the protocol procedure given below. Culture media were refreshed every 2–3 days and the cells were passaged upon reaching 70–80% confluence, using 0.25% trypsin-EDTA enzyme solution (Aldrich, St Louis, MO, USA). Before sub-cultivation, the cells were washed with phosphate-buffered saline (PBS) (Biowest, Nuaillé, France). Cell morphology was observed with the use of an inverted microscope (Zeiss, Oberkochen, Germany). MCC13 cells were seeded in flat-bottom 24-well culture plates (VWR, Radnor, PA, USA) in four biological repeats at a density of 1 × 10^4^ cells/well. The cells were allowed to attach for 24 h before treatment. For the entire experiment the cells were cultured in a humid atmosphere at 37 °C and 5% CO_2_.

For the evaluation of antiproliferative activity of hydroxychalcones (1–3), six human cancer cell lines and one murine normal cell line were employed, including human urinary bladder carcinoma (5637), epidermoid carcinoma (A-431), head and neck squamous cell carcinoma (UM-SCC-17A), melanoma (SK-MEL-3), Merkel cell carcinoma (MCC-13), glioblastoma (A172), the murine embryonic fibroblast cell line BALB/3T3 and human mammary epithelial cells (MCF-10A) as a non-cancerous control. The UM-SCC-17A and MCC-13 cell lines were obtained from the European Collection of Authenticated Cell Cultures (ECACC, Salisbury, UK) by M. Stompor-Gorący (University of Rzeszów, Rzeszów, Poland), while A-431, SK-MEL-3, A172, MCF-10A and BALB/3T3 were purchased from the American Type Culture Collection (ATCC, Manassas, VA, USA). The 5637 cell line was obtained from the Riken BRC Cell Bank. Cells were cultured under standard conditions at 37 °C in a humidified atmosphere with 5% CO_2_. The 5637 line was maintained in RPMI 1640 medium (Sigma-Aldrich, Taufkirchen, Germany) supplemented with 10% fetal bovine serum HyClone (GE Healthcare, Chicago, IL, USA) and 2 mM L-glutamine (Sigma-Aldrich, Taufkirchen, Germany). UM-SCC-17A cells were cultured in Dulbecco’s modified Eagle’s medium (IIET, Wroclaw, Poland) supplemented with 15% FBS and 2 mM L-glutamine. Melanoma cells were grown in McCoy’s 5A medium (Corning Inc., Corning, NY, USA) supplemented with 15% FBS and L-glutamine. Merkel cells were cultured in RPMI medium containing 25 mM HEPES (Gibco, Waltham, MA, USA), supplemented with 15% FBS and 2 mM L-glutamine. A-431, A172, and BALB/3T3 fibroblasts were maintained in Dulbecco’s modified Eagle’s medium supplemented with 10% FBS and 2 mM L-glutamine. MCF-10A cells were cultured in Ham’s F-12 medium (Corning Inc., Corning, NY, USA) with 7.5% horse serum (Gibco Thermo Fisher Scientific Inc., Paisley, UK), 2 mM L-glutamine (Sigma-Aldrich, Taufkirchen, Germany), 10 μg/mL insulin, 10 μg/mL hydrocortisone and amino acids (Gibco Thermo Fisher Scientific Inc., Paisley, UK).

All culture media were additionally supplemented with antibiotics: 100 U/mL penicillin (Polfa Tarchomin, Warsaw, Poland) and 0.1 mg/mL streptomycin (Sigma-Aldrich, Taufkirchen, Germany).

### 4.2. Treatment of Cells

**Figure 10:**
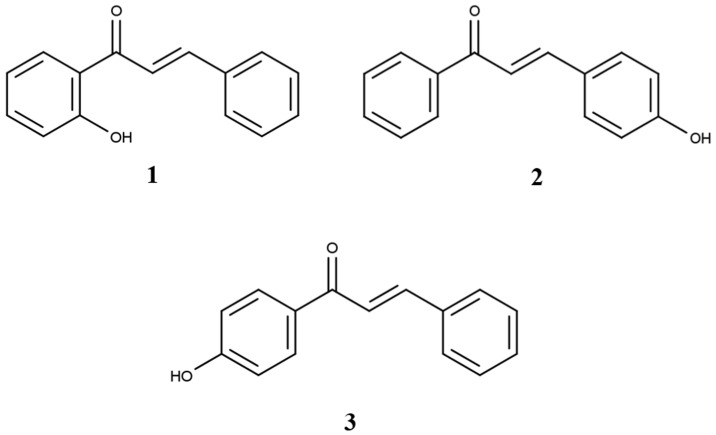
Chemical structures of tested compounds: (**1**) 2′-hydroxychalcone; (**2**) 4-hydroxychalcone (**3**); 4′-hydroxychalcone. Chemical structures were drawn using MarvinSketch 25.5.0 (ChemAxon, Budapest, Hungary).

2′-Hydroxychalcone (C_15_H_12_O_2_, MW = 224.26), 4-hydroxychalcone and 4′-hydroxychalcone, each at the purity of >98% (all of them from Sigma Aldrich, St. Louis, MO, USA) (Figure 10), were dissolved in dimethyl sulfoxide (DMSO) (VWR, Radnor, PA, USA) to produce 100 mM of 2′ hydroxychalcone, 100 mM of 4-hydroxychalcone and 100 mM of 4′-hydroxychalcone stock solutions. The microliter volumes of stock solutions were added to 1 mL of a culture medium to obtain variable concentrations of the studied compounds. The concentration of DMSO was kept at 0.5 *v*/*v*% by the addition of neat DMSO. The control solution consisted of 0.5% DMSO in the medium. The apoptotic tests were performed for 0–100 μM 2′-hydoxychalcone, 0–100 μM 4-hydroxychalcone, and 0–100 μM 4′-hydroxychalcone solutions. The cells were incubated with such media for 24 and 48 h.

Cell lines used for the viability assay were seeded in 384-well plates (Greiner Bio-One, Kremsmünster, Austria) for 24 h before adding tested compounds. Each cell line was seeded in the appropriate media with (5 × 10^3^)–(5 × 10^4^) cells per well. All cell lines were exposed to each tested agent at different concentrations within the range of 0.00001–100 μg/mL, for 72 h. The cells were also exposed to the reference drug cisplatin (ACCORD, Warsaw, Poland). Additionally, all cell lines were treated with dimethyl sulfoxide (DMSO) (the solvent used for dilutions of tested compounds) (POCh, Gliwice, Poland) at the concentrations corresponding to these in solutions of tested agents. After 72 h, a sulforhodamine B (SRB) assay was performed.

### 4.3. The Sulforhodamine B (SRB) Assay

Following 72 h incubation with hydroxychalcones, cells were fixed in situ by the gentle addition of cold 25% trichloroacetic acid (TCA) (POCh, Gliwice, Poland) and incubated at room temperature for 1 h. Subsequently, the wells were washed four times with water and air-dried. A 0.1% solution of sulforhodamine B (Sigma Aldrich, Taufkirchen, Germany) prepared in 1% acetic acid (POCh, Gliwice, Poland) was then added to each well, and the plates were incubated again at room temperature for 30 min. Unbound dye was removed by washing plates four times with 1% acetic acid, whereas remaining dye was solubilized with 10 mM Tris base (Sigma Aldrich, Taufkirchen, Germany). Absorbance was measured using the Synergy H4 Hybrid Multi-Mode Microplate Reader (BioTek Instruments, Inc., Winooski, VT, USA) at 540 nm. IC_50_ values, defined as the concentration of compound required to inhibit cell proliferation by 50%, are presented as mean ± standard deviation. Each concentration was tested in triplicate within a single experiment, and all experiments were independently repeated at least three times.

### 4.4. Determination of Apoptosis by Annexin V Staining

To assess the stages of apoptosis, an Annexin V Dead Cell Kit (Cat. #MCH100105, Merck Millipore, Burlington, MA, USA) was used according to the manufacturer’s protocol. In general, MCC13 cells grown in 24-well plates were treated with increasing concentrations of hydroxychalcones (1–3) and DMSO for 24 and 48 h. Next, the cells were collected and incubated with annexin V protein and 7-AAD indicator for 20 min in the dark, at room temperature. Four cell populations were distinguished using a Muse Cell Analyzer (Merck Millipore, Burlington, MA, USA).

### 4.5. Determination of Changes in Mitochondrial Membrane Potential

To determine changes in mitochondrial membrane potential, cells were conditioned for MitoPotential Assay (Cat. #MCH100110, Merck Millipore, Burlington, MA, USA) as specified by the manufacturer’s protocol. MCC13 cells were treated with increasing concentrations of hydroxychalcones (1–3) and DMSO for 24 and 48 h. Then, the cells were collected, washed and incubated with the Muse Mitopotential Dye for 20 min at 37 °C in the MS-100 Thermo Shaker block (Eppendorf, Hamburg, Germany). Finally, cells were incubated with 7-AAD marker for 5 min at room temperature.

### 4.6. Detection of Oxidative Stress Potential

For the detection of superoxide radicals, the Oxidative Stress Kit (Cat. #MCH100111, Merck Millipore, Burlington, MA, USA) was used according to the manufacturer’s protocol. MCC13 cells were treated with increasing concentrations of hydroxychalcones (1–3) and DMSO for 24 and 48 h. Next, the cells were collected and combined with the Muse Oxidative Stress Reagent working solution by vortexing for 5 s. Finally, the cells were incubated for 30 min at 37 °C in the MS-100 Thermo Shaker block (Eppendorf, Hamburg, Germany), protected from light.

### 4.7. Statistical Analysis

Each of the experiments was performed in four technical replicates per condition and the results of the analyses were presented as medians. The statistical analysis covers ANOVA (analysis of variance), Friedman and post hoc tests. Comparisons between groups were performed with Prism Software 10.4.1 (GraphPad Software, Inc., San Diego, CA, USA). Statistical significance was accepted at *p* < 0.05. The gating was set based on untreated (DMSO) samples to establish thresholds.

## 5. Conclusions

In this work we provided evidence for the anticancer effect of three hydroxychalcones, namely 2′-hydroxychalcone, 4-hydroxychalcone and 4′-hydroxychalcone, on phosphatidylserine exposure, associated with programmed cell death. Generated data in our study revealed that these compounds were involved in mitochondrial membrane activity and redox balance in cells, depending on dose and exposure time. In addition, we explained that the differences in activities of the hydroxychalcones arise from the position of the hydroxyl group, which affects molecular interactions. All compounds showed antiproliferative and pro-apoptotic effects on the MCC13 cell line, with compound 2′-hydroxychalcone exhibiting the highest activity in mechanistic assays. Collectively, these findings provide a proof of concept that structurally related hydroxychalcone isomers are capable of modulating cancer cell viability and apoptosis-related pathways. To summarize, the analyzed compounds exhibit antiproliferative activity based on the generated data, which may indicate potential for further investigation; however, their biological effects may vary and even be reversed, depending on the disease indication. Thorough molecular evaluation of their activity is therefore essential before therapeutic application.

ROS generation and mitochondrial membrane depolarization are often early and dynamic responses to bioactive compounds. As this is an in vitro preliminary study we only focused on the effect on aforementioned cell lines and neither in vivo nor molecular validation was performed at this stage. A more detailed comparative analysis correlating chemical structure with ROS induction patterns, mitochondrial membrane potential changes, and time- or dose-dependent responses may improve the mechanistic insight and justify the inclusion of multiple derivatives in the future study using the different tumor models.

## Figures and Tables

**Table 1:** IC_50_ values of tested compounds on tumor and healthy cell lines.

Compound		IC_50_ [μM]							IC_90_ [μM]
	5637	A-431	UM-SCC-17A	SK-MEL-3	MCC13	A172	MCF-10A	BALB/3T3 Clone A31	MCC13
**(1) 2′-HC**	-	-	-	-	25.7 ± 6.4	-	18.8 ± 2.7	-	61.19
**(2) 4-HC**	15.2 ± 1.1	12.0 ± 1.8	46.2 ± 2.8	52.8 ± 5.4	27.0 ± 7.1	30.3 ± 8.8	43.3 ± 15.5	13.6 ± 4.3	70.63
**(3) 4′-HC**	6.7 ± 2.7	11.1 ± 4.0	27.6 ± 2.8	30.0 ± 8.0	13.9 ± 4.8	16.3 ± 5.2	23.7 ± 5.3	14.4 ± 5.4	36.72
**Cisplatin**	1.67 ± 0.7	4.0 ± 1.3	17.3 ± 4.3	14.0 ± 4.7	7.3 ± 3.3	23.6 ± 7.7	-	1.7 ± 0.7	-

**Table 2:** The selectivity indexes (SI) representing IC_50_ for normal cell line/IC_50_ for cancerous cell line.

Compound	SI_1_/SI_2_					
	5637	A-431	UM-SCC-17A	SK-MEL-3	MCC13	A172
**(1) 2′-HC**	-	-	-	-	0.73/-	-
**(2) 4-HC**	2.84/0.89	3.60/1.13	0.93/0.29	0.82/0.25	1.60/0.5	1.42/0.44
**(3) 4′-HC**	3.53/2.14	2.13/1.29	0.85/0.52	0.79/0.48	1.7/1.03	1.45/0.88
**Cisplatin**	-/1.01	-/0.42	-/0.09	-/0.12	-/0.23	-/0.07

## Data Availability

Data is contained within the article.

## Abbreviations

The following abbreviations are used in this manuscript:

2′-HC	2′-hydroxychalcone
4′-HC	4′-hydroxychalcone
4-HC	4-hydroxychalcone
5637	bladder carcinoma cell line
A172	glioblastoma cell line
A-431	epidermoid carcinoma cell line
BALB/3T3	mouse fibroblast cell clone A31
FXN	protein frataxin
iPSCs	induced pluripotent stem cells
MCC13	Merkel cell carcinoma
MCF-10A	mammary cell line
MMP	mitochondrial membrane potential
NF-kB	nuclear factor-kB
-OH	hydroxyl
ROS	reactive oxygen species
SK-MEL-3	melanoma cell line
TSAHC	4′-(p-toluenesulfonylamido)-4-hydroxychalcone
UM-SCC-17A	head and neck squamous carcinoma cell line
